# Density and Thermal Expansivity of Molten 2LiF-BeF_2_ (FLiBe): Measurements and Uncertainty Quantification

**DOI:** 10.1021/acs.jced.2c00212

**Published:** 2022-11-15

**Authors:** Ricardo Vidrio, Sara Mastromarino, Evan Still, Louis Chapdelaine, Raluca O. Scarlat

**Affiliations:** † Nuclear Engineering Department, University of California Berkeley, Berkeley, California94720-0001, United States; ‡ Department of Nuclear Engineering and Engineering Physics, University of Wisconsin − Madison, Madison, Wisconsin53706, United States

## Abstract

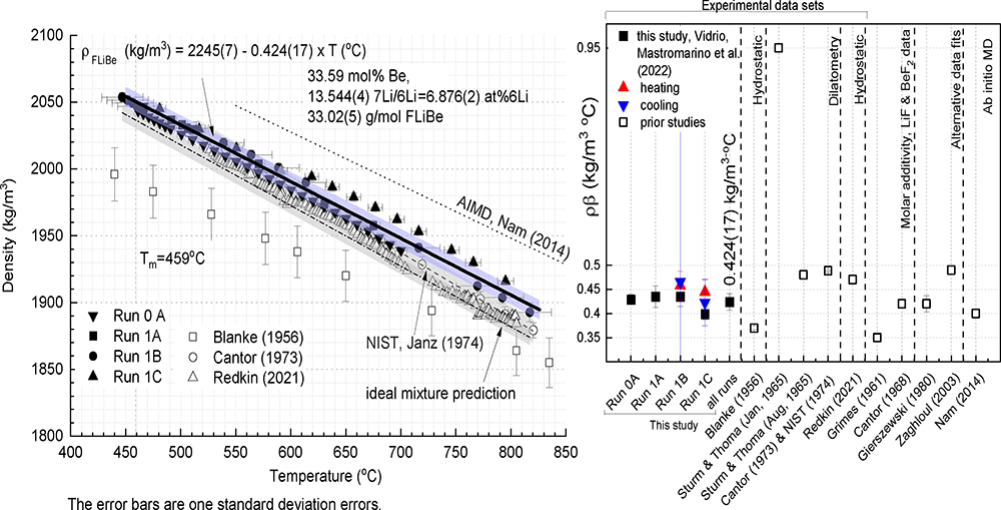

The variability among prior data for FLiBe is 11% for the liquid density and 61% for the thermal expansivity. New liquid density and thermal expansivity data are collected, with particular attention to uncertainty quantification. We discuss and quantify bounds for possible sources of variability in the measurements of liquid density: salt composition (<0.6% per 1 mol % BeF_2_), salt contaminants at 100 s ppm to <1 mol% (2%), Li isotopic composition (2%), sample isothermal conditions (0.2%), dissolved gases (<0.3%), and evolution of bubbles with temperature transients – depending on Ar or He cover gas (0.1 or 0.6% for dilatometry, 1 or 5% for hydrostatic measurements). To aid in quantifying thermal expansivity sensitivity to composition, we review and generalize the ideal molar volume prediction for FLiBe; to improve this model, measurements are needed for the thermal expansivity of BeF_2_. We collect new data on the density of liquid FLiBe using the hydrostatic method and 170 g of hydrofluorinated FLiBe with less than 0.13 mol % impurities (dominantly Al, K, Na, Mg, Ca), as determined by ICP-MS. We obtain the following:  The dominant sources of uncertainty are the bobber volume uncertainty (0.15%), the mass measurement uncertainty (0.2%), and possibly the wetting angle of the salt on the wire (<0.3%). Occasional noise and <0.2% deviation from linearity may be due to the formation of gas bubbles on the bobber surface from the temperature-dependence of gas solubility; repeatable results for heating and cooling runs provide confidence that bubble effects are well managed in this experimental setup. These are the first measurements of the liquid density of FLiBe that report error analysis and that measure the liquid composition before and after density measurements.

## 1 Introduction

The thermo-physical properties of the molten halide salt 2LiF-BeF_2_ (FLiBe) enable inherent safety features and passive safety systems for fusion and fission nuclear reactors that employ FLiBe^1,2,42,43^ therefore characterization of the thermo-physical properties and the corresponding uncertainty quantification is important for safety analysis of nuclear reactors that utilize FLiBe.^44^ For example, Molten Salt Reactors (MSRs) use FLiBe as a solvent for liquid nuclear fuel and fluoride salt-cooled high temperature reactors (FHRs) use FLiBe as a coolant and heat transfer fluid. Both MSRs and FHRs employ natural circulation for decay heat removal, which is a passive safety feature in nuclear reactors. The rate of heat removal in a single phase (liquid) natural circulation loop is proportional to the thermal expansivity (coefficient of thermal expansion multiplied by density) and the elevation difference between the hot and the cold segments of the loop. Thus, the thermal expansivity directly affects the geometric design of this passive safety feature in nuclear reactors, and its error quantification is of relevance to safety analysis of the decay heat removal systems.^45^ As another example of the relevance of density to safety analysis, the density is an input in neutronic analysis which predicts the power and reactivity of the reactor, and the coefficient of thermal expansion is needed in predicting the temperature reactivity coefficient of the coolant in FHRs and of the fuel in MSRs.^45−48^

This study reviews all prior data for the density of FLiBe, discusses the available uncertainty quantification for each data set and quantifies the study-to-study variability. This study explores several possible causes for the study-to-study variability in the liquid density of FLiBe and provides new data for the temperature-dependent density of FLiBe, accompanied by compositional analysis of the salt and uncertainty quantification for the density and thermal expansivity of FLiBe.

## 2 Background

**Figure 1:**
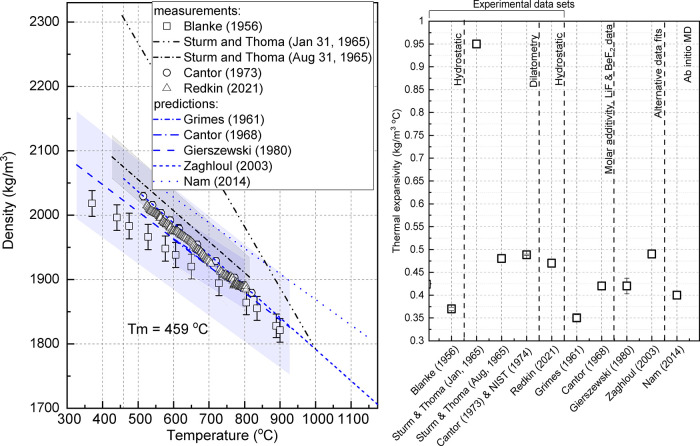
Prior studies for the liquid density and thermal expansivity coefficient of FLiBe: experimental (Table 1) and computational or analytical predictions (Table 2). The error bars for density represent the measurement uncertainty when reported in the literature; the error bar for thermal expansivity are computed based on the reported measurement uncertainty for density across 100 °C. All studies assume constant thermal expansivity with respect to temperature.

While the literature referencing the density of molten 2LiF-BeF_2_ salt (FLiBe) is numerous,^3−6^ the original sources of data for FLiBe liquid density are limited and demonstrate a significant amount of variability. Figure 1, Table 1 and Table 2 summarize all original data and methods for FLiBe density determination. Some of the earliest measurements of high temperature density of molten salts date back to the early 20th century. Jaeger (1917)^9^ measured the densities of fifty molten salts which include nitrates, chlorides, and fluoride salts using the hydrostatic method or buoyant weight method. The method is based on weight measurements of an object immersed in the fluid to be characterized; the density of the liquid can be determined if the density of the immersed object is known. In the mid-1950s the Mound Laboratory used the hydrostatic method to measure liquid density with a similar setup as Janz (that could operate up to 1000 °C under a flow of helium gas) and collected data on several systems: NaF-BeF_2_, LiF-BeF_2_, NaF-BeF_2_–UF_4_, and LiF-BeF_2_-UF_4._^13^ At the Oak Ridge National Laboratory, subsequent measurements were made in the 1950s and 1960s using the hydrostatic method^14^ and the dilatometry method,^12,17,18^ The dilatometry method was operated in an argon glove-box and determines salt volume from measurements of height of the liquid salt sample in a cylindrical containers, using a rod attached to calipers; to determine the position of the salt level, the tip of the rod is part of an open electrical circuit connected to a lightbulb,^14,17^

**Table 1:** FLiBe Liquid Density and Coefficient of Thermal Expansion, in Chronologic Order

	Blanke (1956)^13^	Sturm and Thoma (1965)^18^	Sturm and Thoma (1965)^12^	Cantor (1973)^17,49^	Redkin (2021)^16^	current study, Vidrio and Mastromarino (2022)
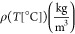	2159(4) – 0.370(6) *T*^a^	2742.00 – 0.95 *T*	2296.00 – 0.48 *T*	2279.7(9) – 0.4900(14) *T*^a^	2390(2) – 0.470(2)*T*^a^	2245 (7) – 0.454 (17) *T*
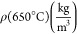	1919(19)	2124.5	1982.8	1962(6)	1955.7	1950 (13)
δρ measurement uncertainty (%)	1%	not specified	not specified	0.3%	not specified	0.3%
δρ fit uncertainty (%)	0.2%^a^	fit uncertainty not specified; data points not provided		0.04%^a^	0.08%^a^	0.1% average residuals
number of data points collected	12	one datum at 648.9 °C and fit equation	one datum at 650 °C and fit equation	11	147 (one run)	61 (four runs)
temp range (°C)	370–900	455–1000	427–816	515–820	527–807	447–817
temp uncertainty δ*T*(°C)	0.5	not specified	not specified	0.2	not specified	4
temperature calibration details	not specified	not specified	not specified	not specified	not specified	NIST-traceable calibration of oven TC; probe vendor calibration only for in-salt TCs
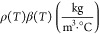	0.370(6)	0.95	0.48	0.4900(14)	0.47	0.454(17)
δρ(*T*)β(*T*) (%)	1.6%^a^	not specified	not specified	0.3%^a^	0.4%^a^	4% (error propagation using York fitting method)
experimental method	hydrostatic	dilatometry	dilatometry	dilatometry^49^	hydrostatic	hydrostatic
details relevant to calibration	measure weight of a Pt bobber attached to a Pt wire immersed in the fluid; known Pt density vs temp	measure with a rod and calipers the salt liquid level; measure salt weight; known geometry and thermal expansion of the cylindrical vessel		not specified	balance was calibrated using 50 g and 100 g calibration weights	scale calibrated per ASTM E898-88 (2013) with ASTM Class 1 weights; bobber volume calibrated with standard density liquids
dominant sources of experimental error	uncertainty in weight and temperature measurements		reproducibility of detecting the liquid level impacted by possibility that small amounts of liquid may adhere to the probe	creep sustained by the vessel, bubbles, and small amounts of salt condensed on the upper neck of the vessel	not specified	uncertainty in bobber volume, weight measurements, and wetting angle on the wire (of relevance to surface tension on the wire); possible bubble formation;
container material	Inconel	not specified	not specified	nickel	not specified	glassy carbon
atmosphere	in a closed container with constant flow of He gas	inert glovebox	inert glovebox	argon glovebox for sample loading; 5 atm argon-gas container for measurements	not specified	Ar positive-pressure glovebox with sensors for oxygen and moisture
sample volume (mL)	136.23	not specified	not specified	not specified	not specified	78.41
LiF-BeF_2_ composition (mol % BeF_2_)	33.33 (also 0 to 55 mol % BeF_2_ and with UF_4_ at 0 to 47 mol %)	34	34	34 (ref (14) also 50.2, 74.9, 89.2 mol % BeF_2_)	34 (also 27 mol % BeF_2_)	33.59 (3) (MW_FLiBe_ = 33.02 (5) g/mol)
^7^Li/^6^Li (molar ratio)	not specified	not specified	not specified	not specified	not specified	13.544(4)
method of analysis for BeF_2_mol %	not specified	not specified	not specified	not specified	not specified	MC-ICP-MS
major impurities	not specified	not specified	not specified	not specified	not specified	100 s ppm: Al, K, Na, Mg, Ca

a Fit error not reported; determined here from linear fit to data.

**Table 2:** Theoretical and Modeling Studies that Predict Liquid Density and Coefficient of Thermal Expansion of FLiBe

value	Grimes (1961)^7^	Cantor (1968)^8^	Gierszewski (1980)^19^	Zaghloul (2003)^20^	Nam (2014)^21^	current study, Vidrio and Mastromarino (2022)
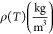	2173.60 – 0.35 · *T*[°C]	2214.00 – 0.42 · *T*[°C]	2215.30 – 0.42 · *T*[°C]	2281.60 – 0.49 · *T*[°C]	2265.60 – 0.40 · *T*[°C]	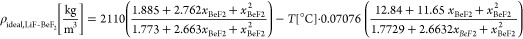 (2220 – 0.430 *T* for FLiBe)
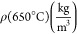	1944.90	1940(40)	1940(80)	1963.10	2005.60	1940.50
uncertainty (%)	2%	2%	4%	not specified	not specified	-
temp range (°C)	600–800	600–800	327–927	459–4225	550–1150	
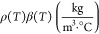	0.35	0.42		0.49	0.40	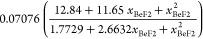 (0.430 for FLiBe)
method	additive law of molar volumes of LiF and BeF_2_	additive law of molar volumes of LiF and BeF_2_	extrapolated linear fit of the FLiBe experimental values from two studies	extrapolating toward critical temperature from Cantor experimental data, using critical temperature and density values that originate from a soft sphere molecular dynamics model^22^	ab initio molecular dynamics (AIMD); (for comparison, AIMD-predicted density is also reported by ref (23): ρ(700°C) = 2100(300) kg/m^3^) vs 1974 kg/m^3^ predicted by ref (21)**)**	additive law of molar volumes
Experimental data employed	LiF from^9^ in the temperature range 868.5 to 1270 °C using the hydrostatic method, BeF_2_ from^11^ at 800 °C	LiF from^10^ in the temperature range 850.4 to 1094.3 °C using the hydrostatic method, BeF_2_ from^12^ at 600 and 800 °C (data source not cited)	(12,17)	(17)	-	(10,12)(see Section 4.1)
LiF-BeF_2_ composition (mol% BeF_2_)	34	34	34	34	33	0–100(33.33 for FLiBe)

Overall, FLiBe density values are reported in the temperature range from 327 to 4225 °C and experimental data is available from 370 °C to 1000 °C; not all studies provide the data points, and none of the experimental studies provide details for error analysis or for salt compositional analysis. We note also that the experimental value at 370 °C reported by Blanke is 89 °C below the melting point of FLiBe; the linearity of density with temperature is implicitly assumed to continue into the supercooled liquid state, but only two data-points are available below the melting point and this assumption is not discussed. The variability in the available data sets is higher than the reported errors for the density and even more pronounced for the thermal expansivity. Molar additivity predictions fall between experimental values for density and on the lower end for thermal expansivity. The AIMD prediction over-estimates density compared to experimental measurements, and the AIMD-predicted thermal expansivity falls between the experimental values. Collection of new data is warranted, with careful attention to instrument calibration and uncertainty quantification.

### 2.1 Comment on the FliBe Density Correlation in the 1974 NIST Compendium

The 1974 National Institute of Standards and Technology (NIST) compendium of molten salt thermophysical properties^24^ cites^25^ for the density of FliBe and reports the following correlation:
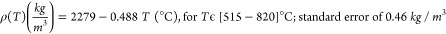
1

Some variability in interpretation and application of the density correlation is observed between the 1974 NIST compendium^24^ and the original publication that it cites^17^: the reported temperature range of validity is different, and the error is reported differently (see Table 1). For example, the density at 650 °C would be 1961.80(46) as per Janz and 1961(6) as per Cantor; the thermal expansivity would be 0.488(5) as per Janz and 0.49(8) as per Cantor (assuming a delta-temperature of 100 °C in the error propagation from density to thermal expansivity). The Janz correlation would be valid at 530 °C, whereas it would be an extrapolation from the available data in Cantor; the Janz correlation would be outside of the range of applicability at 810 °C, whereas it would fall within the range of temperature data collected by Cantor. An explanation for these modifications is not given.^24^

## 3 Experimental Section

The liquid density of FliBe is measured using the hydrostatic method.

### 3.1 Salt Sample Preparation

FliBe was prepared by hydrofluorination by^26^ and,^27,36^ using lithium fluoride and beryllium fluoride (Table 3). An all-nickel transfer vessel is used to transfer salt from the hydrofluorination apparatus to a glovebox. All subsequent sample preparation and storage is performed in a glove box (LC-Technology) with argon atmosphere, with oxygen and moisture below 1 ppm, as verified by glovebox sensors, which operate continuously and are read several times over the course of a day. The molten salt is poured from the transfer vessel onto nickel trays and frozen into chunks that are then stored in glass jars. Frozen chunks are melted in a glassy carbon crucible (SIGRADUR GAT 32 crucible, HTW, Germany) for the purpose of liquid density measurements.

**Table 3:** CAS Registry Number, Supplier, and Mass Fraction of the Chemicals

component	CAS Reg. No.	suppliers	mass fraction
lithium fluoride	7789-24-4	Noah Technologies	99.8%
beryllium fluoride	7787-49-7	Materion	98.8%
1,1,2,2-tetrabromoethane	79–27-6	Cargille Laboratories	60–80%

### 3.2 Salt Characterization

Differential scanning calorimetry (DSC) with a PerkinElmer 800 instrument is performed on the salt prior to density measurements. Powder FliBe is loaded in 0.6-cm hermetically sealed gold pans (TA Instruments); the loading and sealing of the gold pan is performed in the glovebox. Six DSC runs on two samples (12.1 and 13.1 mg) are performed by two different operators over the course of two years. All the runs consist of a heating cycle at 10 °C/min. The DSC is calibrated before the measurement using Zn and In standards.

Inductively coupled plasma mass spectrometry (ICP-MS) is used to analyze salt composition. Following each of the experimental runs, a sample of FliBe is removed from the experimental batch and individually analyzed with ICP-MS. The salt sampling is done in molten state, using a nickel ladle. The ICP-MS analysis is performed by the University of Wisconsin – State Laboratory of Hygiene following the procedure described in.^28^ The major (Na, K, Al, Ca, Mg) and minor contaminants (B, Mg, P, S, Sc, Ti, V, Cr, Mn, Fe, Co, Ni, Cu, Zn, As, Se, Rb, Sr, Y, Zr, Nb, Mo, Rh, Pd, Ag, Cd, Sn, Sb, I, Te, Cs, Ba, La, Ce, Pr, Nd, Sm, Eu, Gd, Dy, Ho, Tm, Yb, Lu, Hf, Ta, W, Re, Ir, Pt, Au, Hg, Tl, Pb, Th, U) are measured by SF-ICP-MS.^28^ The Li/Be and Li-7/Li-6 ratios are measured by MC-ICP-MS on the Neptune Plus (Thermo Fisher Scientific, Bremen Germany).^50^

### 3.3 Density Setup

**Figure 2:**
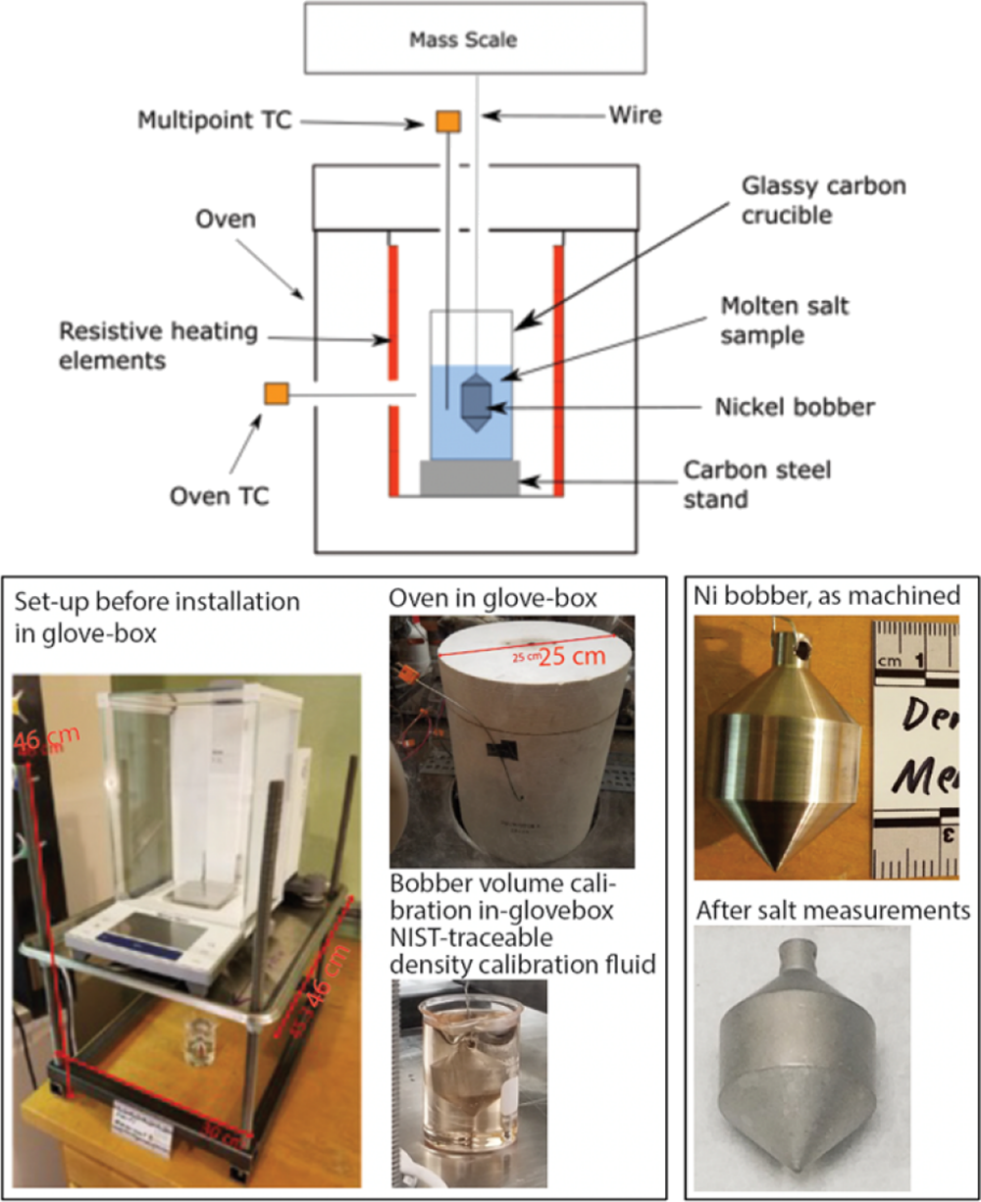
Experimental set-up schematic and photographs for high temperature hydrostatic liquid density measurement.

The hydrostatic density setup (Figure 2) is composed of an analytical scale (Mettler Toledo XS104) equipped with an under-hook and has a maximum weight capacity of 120 g and a minimum readability of 0.1 mg, an oven, and a glassy carbon crucible (Figure 2). The data are manually collected every 30 min in run 0A and continuously logged at 1 Hz in 1A, 1B, 1C. Oven temperature control and temperature recording is performed using a LabView program. The setup is placed in an argon glovebox (LC Technology). The hanging wire is stainless steel 316 L (McMaster Carr), 30 cm long (< 4 cm immersion depth), 0.508 mm diameter. The bobber is machined from 1″(2.54 cm)-diameter bar stock of nickel alloy 200 (onlinemetals.com; nickel 99.0%), 8900 kg/m^3^,^29^ 98.76 ± 0.25 g, 11.149 ± 0.017 cm^3^, with an approximate total height of <4 cm. FliBe is melted in a cylindrical glassy carbon crucible (SIGRADUR GAT 32, HTW Germany), 320 mL, 100 mm height, tapered with 70 mm bottom outer diameter and 73 mm top outer diameter and 3 mm wall thickness; the salt liquid level is 5–6 cm from the bottom. Below the scale is positioned a 1 kW ceramic cylindrical furnace heated through electrical resistance (DS Fibertech), 1200 °C maximum temperature, 10 cm inner diameter, 25 cm inner height, designed with a mullite tube drilled through the side to allow a thermocouple (type N Omega single point thermocouple) to be inserted to control the oven temperature. The furnace lid, has, in the center, three holes of different diameters (the middle one is 1.27 cm in diameter, while the other two have diameters of 0.76 cm) that allow entry for the bobber wire and thermocouples. Either a single-point type N thermocouple or a six-point type K thermocouple array is submerged in the salt for each run. The single-point thermocouples are ungrounded and have a length of 30.5 cm and diameter of 1.6 mm with 1.27 cm spaced points and 0.32 cm diameter stainless steel sheath. For run 0A, a single-point thermocouple is used. For run 1A, the bottom three reading points are submerged in the salt and are recorded, with the bottom tip touching the bottom of the crucible. For run 1B, the multi-point probe is just above the surface of the salt; oven thermocouple measurements for both run 1B and 1A were within 20C of the temperature recorded by the multi-point probe, thus we assume that the readings for run 1B are valid even if the probe is not submerged in the salt and an error of 20 °C is used for the temperature measurments in this run. For run 1C, a single point thermocouple is used; for error propagation, the temperature gradient is conservatively assumed to be

4 °C, the maximum gradient observed in run 1A.

### 3.4 Experimental Procedure

Prior to collecting data, the bobber hangs over the solid salt. After the system reaches 600 °C the salt is molten, and the bobber is placed into the salt. The in-salt thermocouple is then pushed into the sample. Through visual observation, lifting the oven lid, it is confirmed that both the bobber and thermocouple are submerged in the salt. Density measurements are performed at 447–820 °C with 8 to 50 °C temperature increments. For each temperature step, the system is allowed 40 min for the temperature and mass to reach thermal equilibrium. The equilibrium values (the mean of about 600 points collected over the last five minutes of data collection for each temperature step, for both temperature and mass readings) are used for calculating the density. Each experiment runs from 8 to 13 h. The bobber is completely submerged in the molten salt during this time.

Once the experiment is concluded, the oven is turned off and the bobber is lifted from the salt. The setup is cooled for a minimum of 12 h before performing a new experiment. Once the oven is at room temperature the solidified FliBe is weighed and any changes in mass before and after the experiment are recorded. Likewise, changes in the bobber mass, wire mass, and hook mass are recorded (Table 4). Any changes in the mass for the bobber, wire, or hook mass are implemented into the uncertainty calculations.

**Table 4:** Measurements of FLiBe Liquid Density^a^

run details	0A		1A		1B		1C	
starting temperature (°C)	461(3)		467(5)		506(20)		619(6)	
duration of experiment (h)	14(1)		8(1)		13(1)		13(1)	
final temperature (°C)	700(5)		675(6)		467(20)		481(5)	
mass of FLiBe (g)	346.95(1)		347.02(1)		347.29(1)		347.36(1)	
change in the mass of the salt and crucible (g)	(before 0A)		+0.07(1)		+0.27(1)		+0.07(1)	
mass of nickel bobber (g) (*V* = 11.149(17) cm^3^)	98.7615(2) (before 0A)		98.7615(2)		99.7620(2)		99.7642(2) (after 1C)	
bobber mass change (g)			0.0000(1)		+0.0050(1)		+0.0022(1)	
bobber volume (mL)	11.149(17) at 20(2) °C							
wire + hook mass (g)	1.2105(1)		1.2105(1)		1.2072(1)		1.2058(1)	
wire mass change (g)			0.0000(1)		-0.0033(1)		-0.0014	
hook mass change (g) (hook mass = 0.2790(1) g)			0.0000(1)		0.0000(1)		0.0000(1)	

density data		0A		1A		1B		1C	
		T(°C)	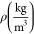	T(°C)	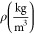	*T*(°C)	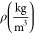	*T*(°C)	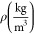
		461 (3)	2043 (3)	467 (5)	2046 (3)	447 (19)	2054 (4)	481 (5)	2045 (3)
		466 (3)	2041 (3)	491 (5)	2037 (3)	452 (19)	2051 (4)	517 (6)	2030 (3)
		471 (4)	2039 (3)	501 (5)	2033 (3)	457 (19)	2049 (4)	551 (6)	2015 (3)
		476 (4)	2037 (3)	511 (5)	2030 (3)	461 (19)	2047 (4)	591 (6)	1998 (3)
		481 (4)	2035 (3)	571 (6)	2004 (3)	506 (19)	2030 (3)	619 (6)	1994 (3)
		486 (4)	2032 (3)	630 (6)	1974 (4)	539 (19)	2020 (3)	638 (6)	1986 (3)
		491 (4)	2030 (3)	675 (6)	1958 (4)	564 (19)	2011 (3)	657 (6)	1979 (3)
		501 (4)	2026 (3)			589 (19)	2001 (3)	677 (7)	1971 (3)
		511 (4)	2022 (3)			614 (19)	1990 (3)	697 (7)	1962 (3)
		521 (4)	2018 (3)			716 (19)	1941 (4)	716 (7)	1953 (3)
		531 (4)	2014 (3)			770 (19)	1912 (5)	746 (7)	1939 (3)
		541 (4)	2010 (3)			792 (19)	1904 (4)	766 (7)	1930 (3)
		551 (4)	2006 (3)			817 (19)	1893 (7)	795 (7)	1916 (3)
		561 (4)	2002 (3)						
		571 (4)	1997 (3)						
		581 (4)	1993 (3)						
		591 (4)	1989 (3)						
		601 (5)	1984 (3)						
		611 (5)	1980 (3)						
		621 (5)	1976 (3)						
		630 (5)	1972 (3)						
		640 (5)	1968 (3)						
		650 (5)	1963 (3)						
		660 (5)	1959 (3)						
		670 (5)	1954 (3)						
		680 (5)	1949 (3)						
		690 (5)	1943 (3)						
		700 (5)	1939 (3)						

	0A	1A	1B			1C		
fitting parameters from York regression	all data (heat)	all data (heat)	all data	heat	cool	all data	heat	cool
ρ (0 °C)	2241 (7)	2250 (12)	2251 (12)	2267 (20)	2262 (400)	2238 (8)	2271 (18)	2248 (26)
ρβ	–0.427 (10)	–0.44 (2)	–0.44 (2)	–0.46 (3)	–0.5(9)	–0.399 (12)	–0.44(3)	–0.42 (5)
data points *N*	28	7	13	9	4	13	9	4
degrees of freedom D.F.	26	5	11	7	2	11	7	2
residual sum of sq. RSS	1.07	0.888	2.06	0.657	4.5E-4	6.51	0.465	3.4 × 10^–5^
correlation coefficient *r*	–0.9997	–0.9990	–0.9980	–0.9988	–0.9992	–0.9971	–0.9993	–1
coeff. of determination *R*^2^	0.9994	0.9979	0.9950	0.9975	0.9984	0.9943	0.9981	1

a Ambient pressure in the argon glovebox is 102(1) kPa. All error bars are provided in parentheses and indicate measurement standard uncertainty.

After each run, a part of the salt is sampled, while molten, and stored for compositional analysis. The rest of the salt is weighed again before starting a new run of measurements. The bobber volume, the scale and one of the thermocouples are NIST calibrated before starting the density measurements (calibration details are given in Section 3.6).

### 3.5 Beryllium Safety

The glovebox, fume-hood, and personal protective equipment provide protection from respiratory and dermal exposure to beryllium-containing chemicals. Beryllium contamination in the laboratory is monitored by surface swipes of the laboratory floors and benchtops. The experimental work was performed from March 2017 to December 2019 during which over ten surface swipes were analyzed. Any detection of beryllium above the detection limit of 0.025 μg/100 cm^2^ (two swipe samples with detectable Be) is followed by cleaning and decontamination procedures. The housekeeping goal for the laboratory in which this work was performed is 0.2 μg/100 cm^2^.

### 3.6 Calibration

#### 3.6.1 Analytical Balance

The calibration of the scale is performed inside the glovebox and follows an ASTM E898–88 (2013) top loading scale calibration procedure using calibration masses of 10, 20, 30, 50, and 100 g classified as ASTM class 1. Weights of increasing increments of 10% are added until the scale reads full or nearly full capacity. Weight measurements are recorded and compared against the tolerance specified by the scale manufacturer. The scale linearity was within the 0.2 mg tolerance specified by the manufacturer.

#### 3.6.2 Bobber Volume

The bobber volume is calibrated in the glovebox using the hydrostatic method with two NIST traceable high density anhydrous organic liquids (Cargille Laboratories Precision-Calibrated Heavy Liquids Organic Series, Lot # 050393) with density 2.00 ± 0.005 g/cm^3^ (Cat. No. 12420) and 3.00 ± 0.005 g/cm^3^ (Cat. No. 12450), certified in the temperature range of 15 to 35 °C. The complete chemical composition of the liquids is proprietary; the chemical information released by the supplier is reported in Table 3.

Forty minutes are allotted to the bobber to reach isothermal conditions. A thermocouple is used to verify isothermal conditions within the liquid by moving it to multiple points on the bobber surface. Isothermal conditions are considered satisfactory if no two points on the bobber are more than 2.2 °C apart, as this value corresponds to the systematic error of the thermocouple at room temperature. Throughout the entirety of the volume calibration procedure, all points on the bobber surface (the topmost, middle, and bottom regions) are well within isothermal conditions, with a maximum spatial and temporal variability of 1.1 °C. The bobber submerged weight is recorded, and the volume is calculated to be 11.149 ± 0.017 cm^3^ at the reference temperature of 20(2) °C.

The bobber geometry and mass did not change after the measurements. The bob is weighed before and after each experiment, and no change in mass is observed (Table 4). Visual inspection shows no change in geometry (Figure 2).

#### 3.6.3 Thermocouples

Single-point ungrounded type N thermo-couples (Omega) and one multi-point type K thermo-couples are used. A dry-block TC calibrator (Ametek CTC 1200A Dry Block Calibrator) is used to calibrate the thermocouple responsible for regulating the furnace temperature; the dry-block calibrator is calibrated by Ametek in January 2017, using the NIST traceable instruments: 62776–83, B7112020, 62776–134. The single point and multipoint thermocouples submerged in salt are calibrated by Omega.

#### 3.6.4 DSC Instrument

**Figure 3:**
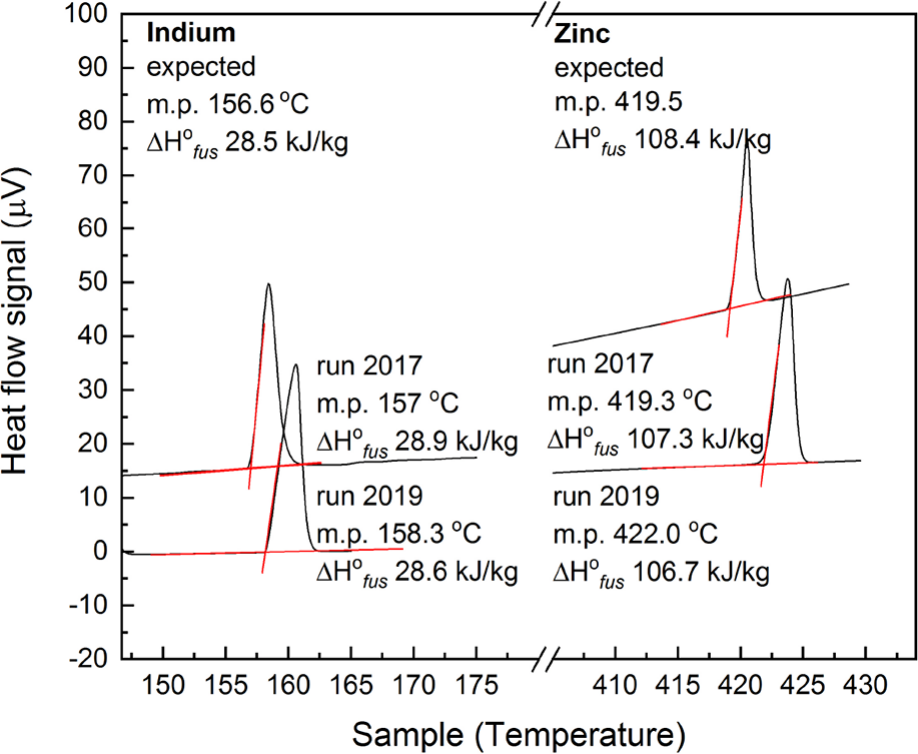
Results for DSC calibration with Zn and In.

The calibration of the DSC is performed measuring two metals with known melting point: indium and zinc. The melting point from literature is compared to the experimental melting point shown in Figure 3 as the onset of the DSC curve. The latent heat from literature is also compared to the experimental latent heat calculated integrating the experimental peak from the DSC.

The data on the In and Zn standards indicates that the measurement uncertainty on the melting point of the salt may be higher than the scatter observed among the six runs on FLiBe (0.2 °C). A 2017 run of a Zn standard shows a deviation of 0.2 °C from the expected Zn melting point of 419.54 °C. A 2019 run of a Zn standard shows a deviation of 2.5 °C. It is unclear if this last high deviation is due to a variation in the operational parameters, or a correct representation of a drift in the measured temperature of the instrument. In the paper, we proceed with reporting the 0.2 °C standard deviation, because we have confidence in the methods and instrument parameters used in collecting this data and we do not have the same confidence for the running of the standards.

## 4 Data and Statistical Analysis

### 4.1 Experimental Data

**Figure 4:**
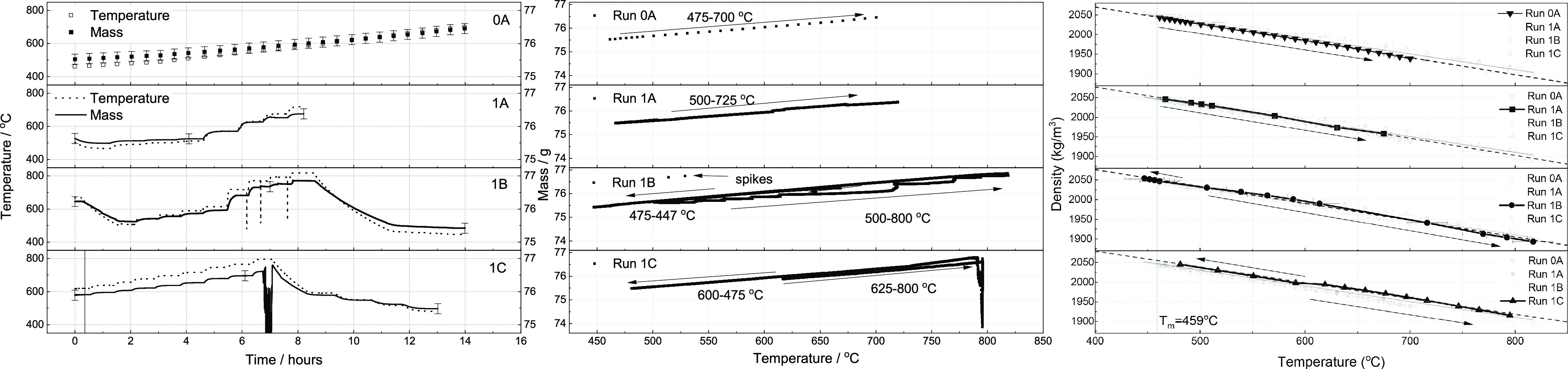
Measurements of FLiBe liquid density.

FLiBe density as a function of temperature is measured during four experimental runs. The results are given in Table 4 and Figure 4. Runs 1B and 1C are collected both following heating and cooling transients; 0A and 1A are collected after heating transients only. In run 1B, three data points are collected below the 459.1(2) ^o^C melting point of FLiBe.

Noise in mass measurements is occasionally observed: in Run 1A the submerged mass measurement displays some noise in the sixth temperature ramp; Run 1B displays some noise during collection of the sixth through ninth data points; when the furnace reaches approximately 800 °C, Run 1C experiences noise in the mass reading that persists for approximately 20 min and disappears on its own. The occasional deviations from linearity (<0.2% residual) of sequential data points are illustrated in Figure 4: for Run 1C, there is an offset in the linear relation between the points measured during heating and cooling. *We postulate that the occasional noise and slight deviation from linearity is due to the formation of gas bubbles on the bobber surface*. Similarly, in^15^ higher data scatter is observed above 670 °C in hydrostatic density measurements for 5LiF–58NaF–27BeF_2_ and it is attributed to bubble formation; bubble removal is similarly discussed in the dilatometry measurements by.^17^ Two bubbles with the diameter of 1 mm will increase effective bobber volume by 0.1%, increasing the measured density value. In future set-ups, visual observations, if achievable, could help verify the absence of bubbles adhered to the bobber surface; in this study, *repeatable results for heating and cooling runs provide confidence that bubble effects are well managed in this experimental set-up* (Section 4.3). Another possible cause for noise and for deviations from linearity is contact of the wire with the inner walls of the hole in the oven lid, changing the mass reading and at the same time possibly introducing oven insulation dust in the salt sample.

### 4.2 Data Reduction and Error Analysis

The molten salt density, , is calculated from *V_bob_* the volume of the bobber, *M_bob_* the weight of the bobber in the gas atmosphere, and *M_sub_* the weight exerted by the bobber when submerged in molten salt. *M_sub_* corrects for the surface tension of the liquid exerted on the wire:
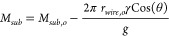
2where *M*_*sub*, *o*_ is the measured weight when the bobber is submerged in molten salt,

*r_wire_* = 0.508 mm is the radius of the submerged wire, γ is the surface tension of the liquid, θ is the contact angle that the liquid makes with the submerged wire, and g is the gravitational acceleration. The surface tension of FLiBe^34^ is:
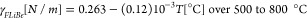
3

A surface tension of 0.20 N/m and a conservative contact angle of zero (fully wetting)^35^ adds a force corresponding to 0.3% (0.07 g) to the mass difference (> 21 g) and is accounted for in the data reduction. With an assumed 30% uncertainty for γ, the uncertainty contribution from surface tension is negligible (< 0.1% added uncertainty to the density measurement). The wetting angle uncertainty (ranging from wetting to nonwetting) can contribute with at most 0.3% uncertainty to the density measurement.

*V_bob_* accounts for the thermal expansion of the nickel, and the submerged wire volume:
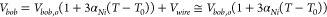
4where *V*_*bob*, *o*_ = 11.149 (17) cm^3^ is the volume of the bobber at *T*_0_***=***20(2)°C, α_*Ni*_ is the coefficient of thermal expansion for nickel, *V_wire_* is the immersed volume of the wire, and *T* is the temperature of the bobber. *V_wire_* = 0.007 cm^3^ or 0.07% of the bobber volume, hence negligible. The temperature-dependent linear coefficient of thermal expansion of nickel^33^ is:
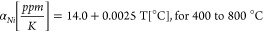
5

The thermal expansion of the nickel bobber is 3.8% from 20 °C to 820 °C. The uncertainty on nickel thermal expansivity is 2% and it contributes 0.08% to the bobber volume uncertainty and is accounted for in the error propagation along with the uncertainty on temperature measurement.

In summary, error propagation accounts for uncertainties in measured parameters (masses and temperature), and data reduction input parameters (bobber volume and temperature-dependent coefficient of thermal expansion and the contributions from wire surface tension). The dominant sources of uncertainty are the bobber volume uncertainty which is 0.15%, the mass measurement uncertainty (+/– 30 mg) that contributes 0.2% uncertainty to the mass difference, and the wetting angle of the salt on the wire which can contribute to up to 0.3% uncertainty to the mass difference.

### 4.3 Regression Method and Analysis of Repeatability

The York method is utilized for data fitting,^30^ to capture error propagation in both the dependent variable X (temperature) and the independent variable Y (density), and the results are given in Table 4 alongside the general fit statistics. In prior literature^18^ the determination of the density and thermal expansivity from density (Y) and temperature(X) data was accomplished via simple linear regression by ordinary least squares (OLS), which provides a closed form solution to the linear fitting *y_i_* = β_1_*x_i_* + β_0_ + ϵ_*i*_. However, OLS cannot inherently account for the uncertainty of the provided data. For data with only error in Y, weighted least squares (WLS) can account for error via a weighting function, which is either equal to the error of each term for direct weights or the inverse of the squared error for instrumental weights. Therefore, the York method is employed here,^30^ using the OriginPro Software (2021, v 9.8). Furthermore, this method will place less emphasis on points with high uncertainty. The error correlation coefficient is assumed to be zero, however a small degree of correlation between the error in X and the error in Y would be expected as the inputs in the data reduction calculations are dependent on temperature.

Under the assumption that the difference between two regression coefficients follows a normal distribution, the Welch’s t-test^31,32^ is selected for performing comparisons between data subsets because it can account for unequal samples sizes, N_1_ and N_2_, and variances by scaling according to the sample size. The number of samples sufficient for application of the central limit theorem is ill defined, but sizes of 30 or 50 are often cited; the approximate degrees of freedom ν to determine the critical value of t^32^ is:
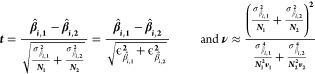
6where β̂_0_, β̂_1_ are the y-intercept and slope of the best fit line, σ_β̂_0__, σ_β̂_1__their standard deviations, ϵ_β̂_0__, ϵ_β̂_1__their standard errors.

To check for repeatability, four experimental runs (0A, 1A, 1B, 1C) are performed on four different dates by two different experimentalists; results of the statistical analysis by the Welch’s two sample t-test (Table 5) with a statistical significance of 0.05 indicate that, *the four runs have no statistically significant difference in the reported thermal expansivity or density*. To check for latent effects of temperature transients, data points are collected both after heating and after cooling temperature steps during runs 1B and 1C. *There are no statistically significant differences in the regression coefficients when comparing post-heating and post-cooling measurements*.

**Table 5:**
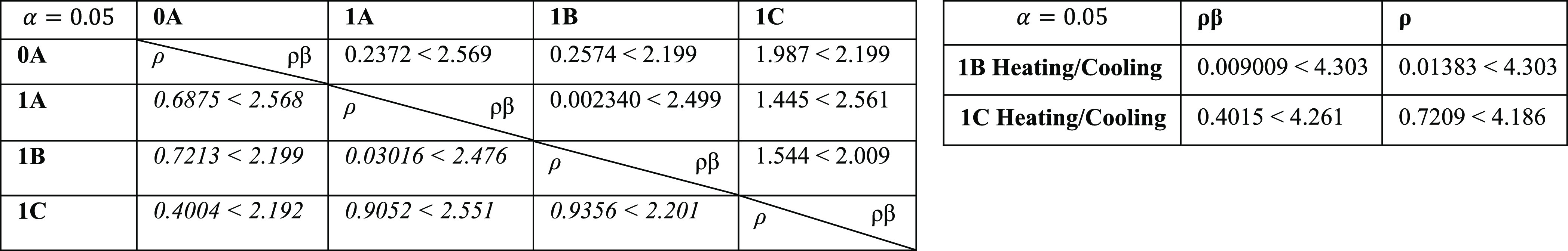
Two-Tailed Welch’s *t* Test Comparison for Density and Thermal Expansivity from Several Data Subsets^a^

a |*t*| > *t*_crit_ would indicate a statistically significant difference; all the subsets show repeatable results.

### 4.4 Density Results

**Figure 5:**
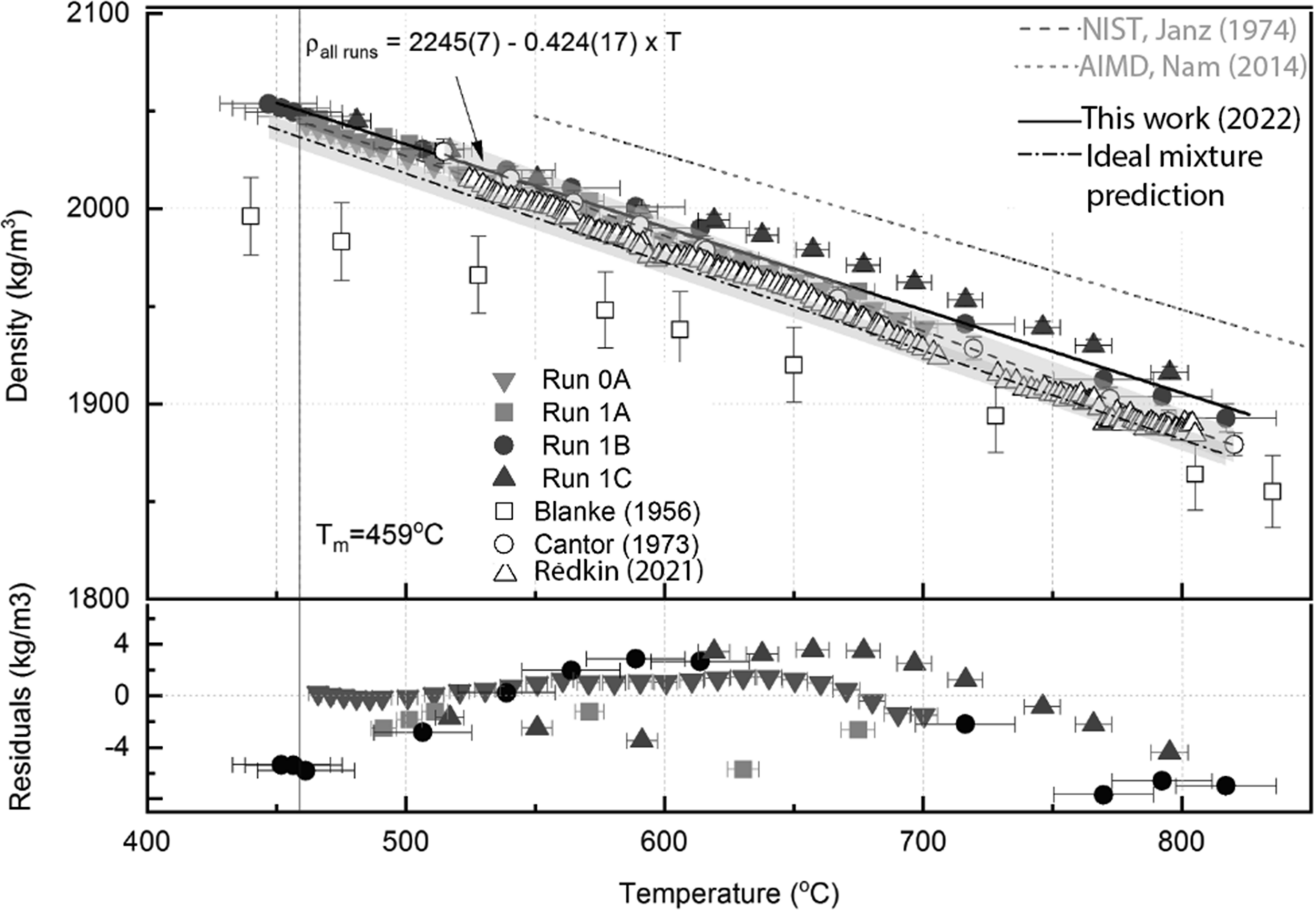
Liquid density measurements for FLiBe. One standard error is reported for the linear fit parameters and it incorporates fit uncertainty and measurement uncertainties in temperature and density. Average fit residual is 0.1%.

Figure 5 shows the data collected for the liquid density of FLiBe. Measurements are performed between 447 °C to 820 °C. The linear fit residual is 0.10%. The overall density correlation ρ_*all runs*_ consists of the averaged parameters from the linear fits of each the four runs: ρ_*FLiBe*_=2245(7) – 0.424(17) T [^o^C]. The measured values of density are within 0.4% of the values measured by dilatometry by^17^ and within 2.7% of the hydrostatic measurements made by,^13^ and 7.3% below that of.^18^ The excess molar volume is −1.5%.

### 4.5 Thermal Expansivity Results

**Figure 6:**
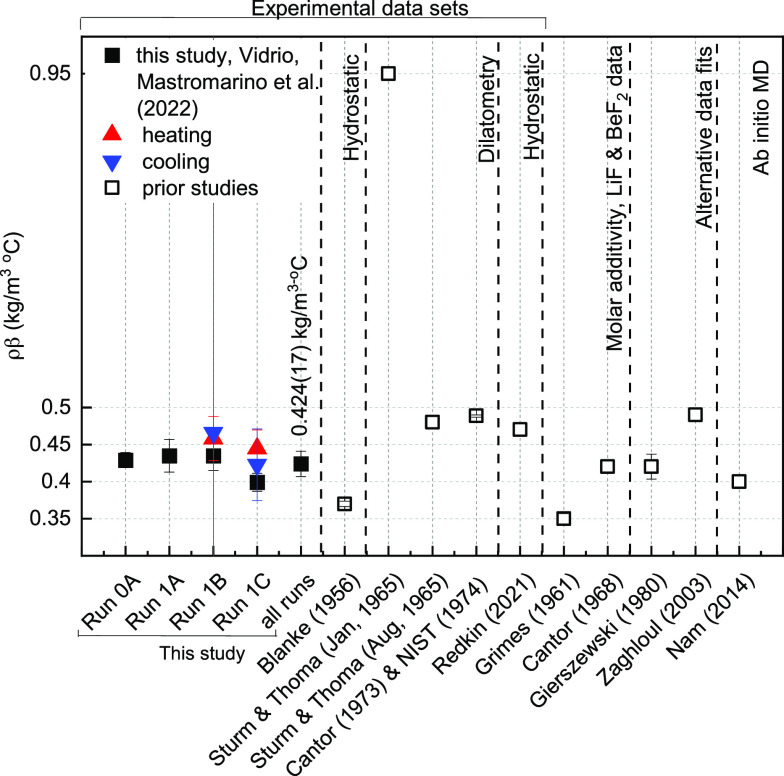
Thermal expansivity of FLiBe. Error bars for this study propagate temperature and density measurement error and fit uncertainty; error bars for prior literature values are computed based on the reported measurement uncertainty for density across 100 °C. All values assume constant thermal expansivity with respect to temperature.

Figure 6 shows the results for the thermal expansivity of FLiBe. The density vs temperature data is generally well-characterized by a linear fit (Figure 4, Table 5) and the thermal expansivity is calculated from the linear fit of the density data. The thermal expansivity of all runs is calculated as the average of the four runs. The thermal expansivity is 13% higher than the hydrostatic measurements of,^13^ 15% lower than the dilatometry measurements of,^17^ 124% lower than in,^18^ and within 1% of the thermal expansivity predicted by molar additivity of LiF and BeF_2_.

### 4.6 Elemental Analysis

**Figure 7:**
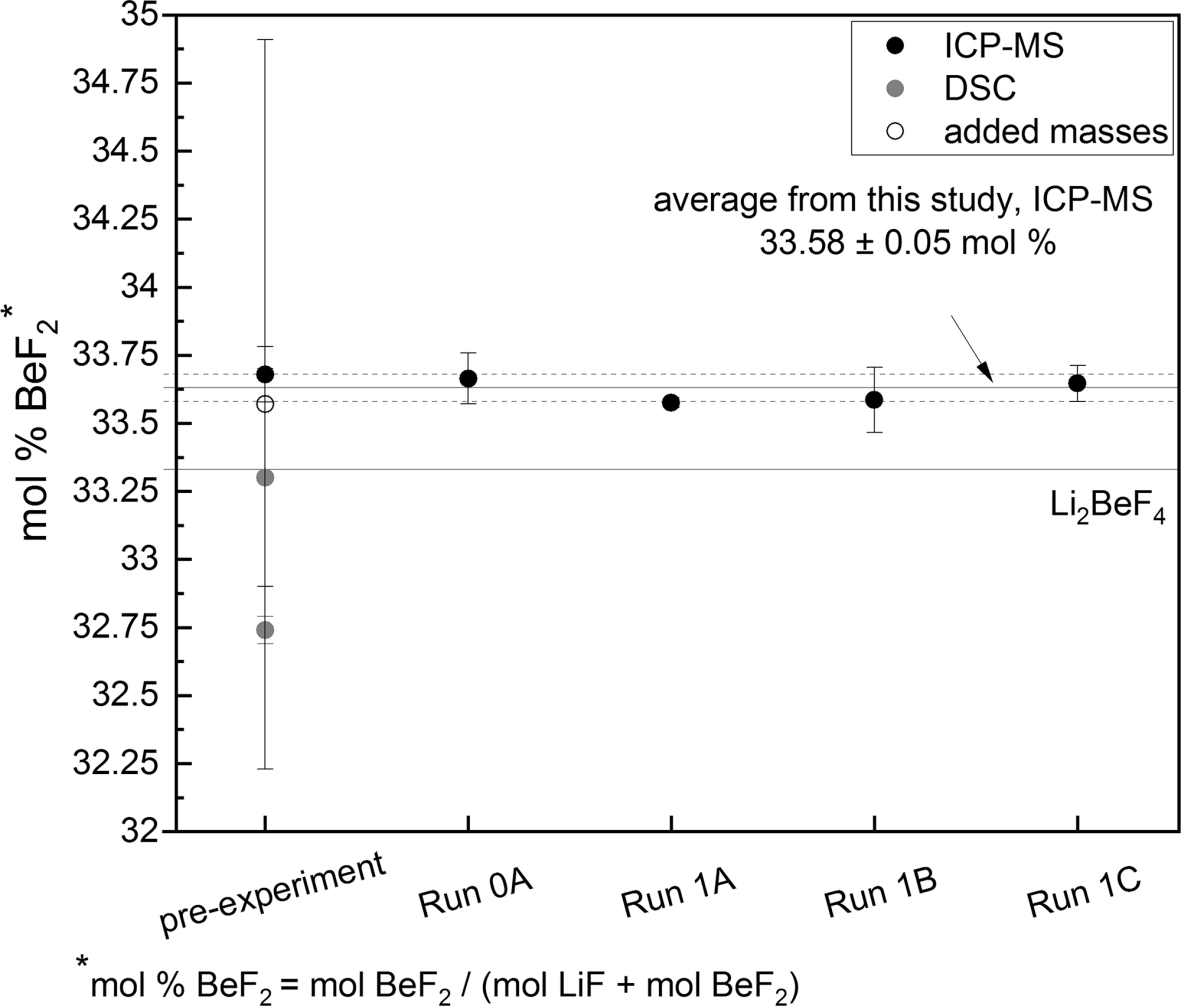
Salt composition of FLiBe sampled before density measurements and after each run. The salt composition before the density measurement is measured by added mass calculation, DSC, and ICP-MS. From the DSC measurement of the melting point, the composition is calculated based on the phase diagram of.^37^ No deviation in salt composition is observed over the course of the experimental runs. The uncertainty is reported as one standard deviation among three ICP-MS runs on one digest; the error on the average composition across all samples is based on error propagation from each point.

Figure 7 shows the mol % of BeF_2_ before and after the density measurements. Before the density measurements, the salt composition is measured using three methods: by weighting the LiF and BeF_2_ added to prepare the FLiBe mixture as reported in,^36^ by DSC measurements and by ICP-MS. The average and standard deviation of the melting point from six DSC runs are 459.1 ± 0.2 °C. After the density measurement, the mol % of BeF_2_ is measured by ICP-MS. We conclude that no deviation in salt composition is observed over the course of the experimental runs, remaining at 33.58(5) mol% BeF_2_. The Li-7/Li-6 isotopic molar ratio is measured to be 13.544(4) (i.e., 6.876(2) at% ^6^Li) for all samples. This corresponds to a FLiBe molecular weight of *MW_FLiBe_* = 33.02 (5) *g*/*mol*.

Table 6 gives the major contaminants in FLiBe: K, Na, Ca, Mg, and Al. Aluminum was the only major contaminant that increased from 99(10) ppm to 270(80) ppm between run 1A and run 1B; all other contaminants remained unchanged (within three standard deviations) over the four runs. The origin of K, Na, Mg, and Ca at 100 s wppm levels in this order of decreasing concentration are original impurities in the raw materials that would not be removed by the hydrofluorination purification process for FLiBe, as reported also in.^51,52^ The origin of the increasing Al contamination is likely the thermal insulation of the oven; when the oven lid is operated, insulation dust can fall into the salt crucible or on the sampled salt. Assuming molar volume additivity (Table 7) we calculate the effect of these dominant impurities on the density of the salt. Comparing 0 ppm impurities to an upper bound of 500 wppm KF, 300 wppm AlF_3_, 300 wppm NaF, 200 wppm MgF_2_, and 150 wppm of CaF_2_, we calculate 0.06% density increase overall with 0.03% density increase from aluminum alone, 0.2% increase in thermal expansivity overall, and with *0.14% increase in thermal expansivity from aluminum alone*. *The density and thermal expansivity variabilities introduced by impurities are smaller than the 0.3% measurement uncertainty of the data reported here.*

**Table 6:** Elemental Analysis of FLiBe before and after Liquid Density Measurements^a^

	ref	0A	1A	1B	1C
K	330(20)	358(18)	339(1)	400(40)	440(50)
Na	278(18)	291(6)	250(3)	252(9)	270(7)
Mg	162(12)	175(1)	154(6)	146(1)	163(3)
Ca	131(6)	142(3)	126(5)	126(4)	133(3)
Al	39(6)	59(8)	99(10)	270(80)	310(60)
structural metals: Cr, Fe, Ni, Mn, Mo, Zr, Ti	34(6)	44(5)	78(7)	72(3)	61(4)
anions: S, P, B, Te, I	8(1)	7(1)	8(1)	8(1)	7(2)
others: Rb, U, Cs, V, Y, As, Cu, Zn, Th, Hf, Sc, Ce, W, Nb, Pd, La, Au, Sb, Co, Sn, Nd, Ag, Ta, Yb, Cd, Pr, Dy, Pb Tl, Gd, Eu, Sm, Lu, Hg, Pt, Ho, Ir, Tm, Re, Rh	32(5)	30(4)	45(7)	26(3)	26(3)

a Error bars in parentheses are one standard deviation of three ICP-MS runs of two digests and six ICP-MS runs.

**Table 7:** Temperature-dependent molar and Ionic Volumes for LiF and BeF_2_ and Possible Contaminants^7,10−12^

	600 °C cm^3^/mol	800 °C cm^3^/mol	ρβ kg/m^3^K
Molar Volumes^12^			
LiF^a^	13.41^c^	14.14^c^	0.51
NaF	19.08	20.2	0.61
KF	28.1	30.0	0.65
RbF	33.9	36.1	0.94
CsF	40.2	43.1	1.27
BeF_2_^b^	23.60	24.4^d^	0.33
MgF_2_	22.4	23.3	0.54
CaF_2_	27.5	28.3	0.40
SrF_2_	30.4	31.6	0.78
BaF_2_	35.8	37.3	0.98
AlF_3_	26.9	30.7	1.93
ZrF_4_	47	50	1.07
ionic volumes^e^			
Be^2+^	assumed negligible molar volume^7^		
Li^+^	1.61	1.94	
F^–^	11.8	12.2	

a Density from^10^ in the temperature range 850 to 1050 °C (using the hydrostatic method).

b Values reported in^12^ (original source not cited).

c Extrapolated values.

d Measured at 800 °C by ref (11) using the hydrostatic method.

e ***V***_***F*^–^**_(***T***) **= *V***_***BeF*_2_**_( ***T***)/**2and *V***_***Li*^+^**_(***T***) **= *V*_*LiF*_**(***T***) **– *V***_***F*^–^**_(***T***)***.***

## 5 Discussion

### 5.1 Molar Volume Additivity

**Figure 8:**
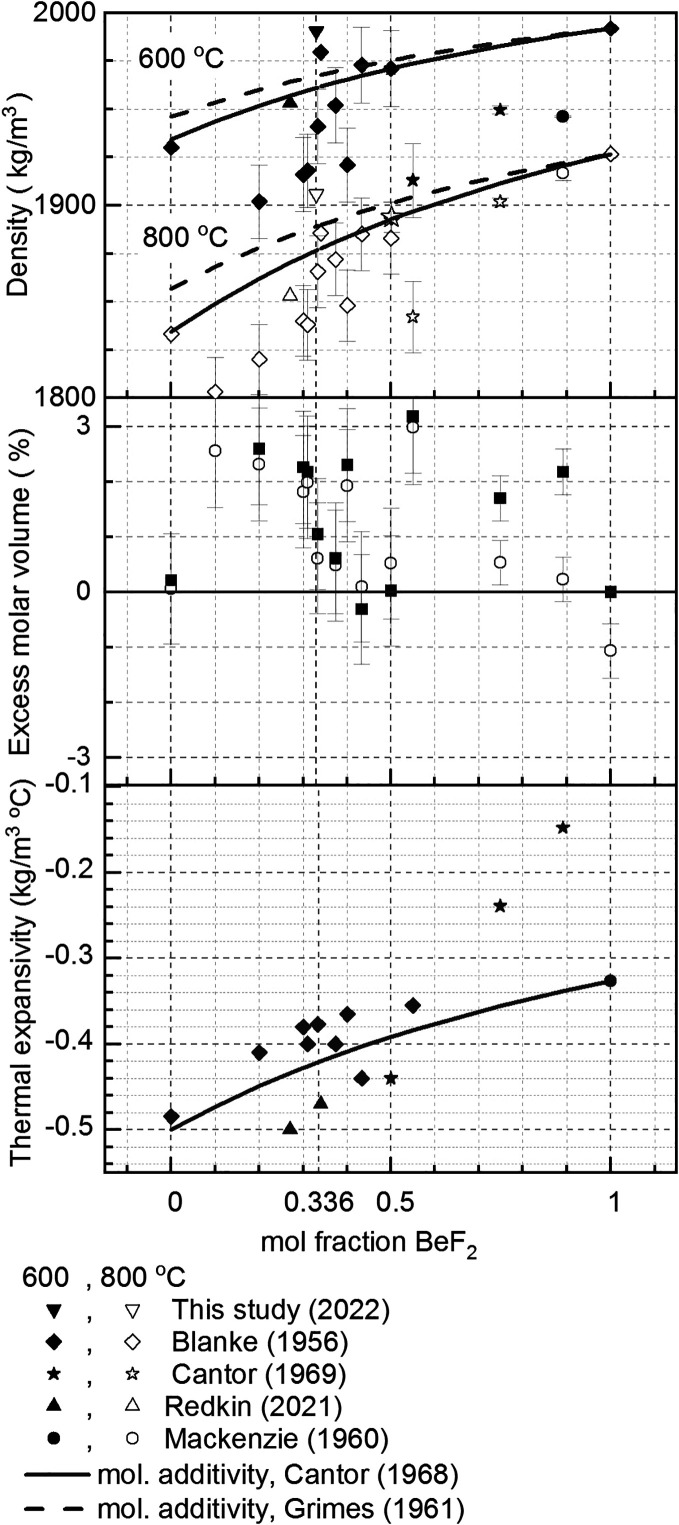
Density, excess molar volume and thermal expansivity for the LiF-BeF_2_ binary mixture. Data from refs (7), (8), (11), (13), (14), (16), and (38). Error bars shown are measurement error as reported by the original data source.

The sensitivity to BeF_2_ concentration is of importance to the error analysis. Figure 8 compiles the available compositional-dependent data and compares it to the molar volume additivity law for ideal mixtures:
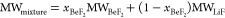
7
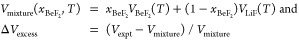
8
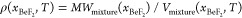
9where *T* is temperature, *x_i_* is the molar fraction of *i*, *MW_i_* is the molecular mass of *i*, and *V_i_* is the molar volume of *i* as a function of the temperature *T*, Δ*V*_excess_ is the excess molar volume of mixing, and *V*_expt_ is the experimentally measured molar volume of the mixtures, and *V*_mixture_ is the molar volume of the ideal mixture. The two studies that have used the molar volume additivity,^7,8,38^ rely on two data sets for the measurement of LiF density vs temperature^9,10^ and one (or possibly two, but original data are not found in the reference report provided) measurement(s) of BeF_2_ density.^11,12^ Using the most recent measurements by^10,12^ and linearizing with respect to temperature, we arrive to an ideal mixture density of:
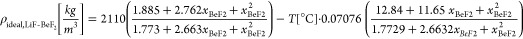
10awhich reduces to a form close to the equation provided by^8^ for the ideal-mixture density of FLiBe (33.3 mol % BeF_2_): . This predicts a compositional sensitivity at 800 °C and 33 mol % BeF_2_ of +0.6% density change per mol% BeF_2_ and + 0.5% thermal expansivity change per mol% BeF_2_. Since the density and thermal expansivity show quite a bit of scatter around 33 mol % BeF_2_, these could be lower-end estimates, and the local slopes with composition could be higher positive values or even negative values.

The excess molar volume is a few %. Thus, for the purpose of the absolute value of density, the molar volume approximation may suffice, and this approached is used in Section 5.2 to quantify the effects of compositional variation. On the other hand, the measured thermal-expansivity can be as much as 140% of the predicted ideal-mixture values for mixtures rich in LiF or as low as 60% of the ideal-mixture values, and the compositional dependence is non-monotonic. Thus, while the ideal mixture assumption provides a starting point estimate, FLiBe is not an ideal mixture and measurements are needed to capture its non-ideal mixture effects. We note also that only one experimental datum of the density of BeF_2_ has been reported to date. The origin of the data point at 600 °C reported in^12^ is not provided, but it leads to a thermal expansivity of 0.33 kg/m^3^ °C for BeF_2_; as a point of comparison, this value leads to over-estimations of mixtures thermal-expansivity compared to measured values: 0.40 for NaF-50% BeF_2_; 0.35 for KF-50% BeF_2_; 0.50 for RbF-50% BeF_2_, 0.41 for NaF-35%LiF-38%BeF_2_^7^ and 0.45 for NaF-LiF-BeF_2_ eutectic.^15^ New measurements are warranted for the thermal expansivity of BeF_2_ to improve the ideal-mixture prediction for the density of the LiF-BeF_2_ binary solution.

### 5.2 Discussion of Sources of Variability in the Density and Thermal Expansivity of Molten FLiBe

The variability in the available data sets is higher than the reported errors for the density and even more pronounced for the thermal expansivity. For example, the highest variation in density is 11% at 600 °C between^13^ and,^18^ and the corresponding variation in thermal expansivity is 61%. The errors for the experimental data are reported only by^13^ and^17^ and are 1% and 0.3% respectively, lower than the 2.2% variability between these two data sets. It would therefore be worthwhile to investigate the sources of variability in the data sets. We postulate here a series of effects that may influence experimental measurements.

A variation in the specific composition of the salt after the experiments might have influenced the results. Elemental analysis of the salt before and after density measurement was not performed for any of the density studies. A change in BeF_2_ composition by 1 mol % would cause a change in density of 0.1% at 600 °C and 0.6% at 800 °C (see Section 5.1 discussion and^38^) and a change in thermal expansivity of 0.5%. Major contaminants may influence the density; assuming molar volume additivity, 500 ppm CsF and 1 mol % ZrF_4_ would lead to 2% density increase of the melt,^39^ and 1 mol % dissolved BeO would lead to 2% density decrease. The isotopics of Li would impact density; F^6^LiBe would have 2% lower density than F^7^LiBe.

It is not reported in any of the FLiBe experimental measurements if isothermal conditions in the experimental setups were reached; for example, a temperature error of 10 °C would lead to a change in density of 0.2%^13^ or 0.5%.^18^ In room-temperature liquids, it is known that dissolved gases may change the density of the liquids; for example, CO_2_ gas dissolved in sulfolane at a concentration of 9.4 × 10^–5^ mol/cc leads to a 0.3% decrease in the density of the liquid at 90 °C.^40^ In the FLiBe experimental studies reported here the cover gas is He, Ar, or not reported; at 1 atm, the solubility of He in FLiBe is 11.5(4) × 10^–8^ mol/cc atm at 600 °C and 19.48(1) × 10^–8^ mol/cc atm at 800 °C; the solubility of Ar in FLiBe is 0.98 (2) × 10^–8^ mol/cc atm at 600 °C and 2.66 (6) × 10^–8^ mol/cc atm at 800 °C.^41^ Even though the gas solubility of He is one order of magnitude higher than that of Ar, the solubility is much lower than in the example given for sulfolane and we would expect an impact of dissolved gases on density well below 0.3% for both He and Ar.

Some of the studies make reference to the presence of bubbles in the molten salt during measurements of the liquid density;^17^ since gas solubility is temperature-dependent, gas evolution is expected with temperature ramp-downs, and hence bubble formation. Both the volumetric and the hydrostatic methods are sensitive to bubble formation. If we consider a sample of 170 g salt (85 cm^3^ salt) at 800 °C, with He cover gas, 0.47 cm^3^ of He gas (7.5 × 10^–6^ mol He) will evolve upon cooling to 600 °C; this corresponds to evolution of 900 gas bubbles of 1 mm in diameter occupying 0.6% of the salt volume (relevant to dilatometry measurements) and 5% of the bobber volume (relevant to hydrostatic measurements with a 10 cm^3^ bobber and assuming that all bubbles nucleate and remain attached to the surface of the bobber). With Ar cover gas, cooling from 800 °C to 600 °C would lead to evolution of 0.10 cm^3^ of Ar gas (1.40 × 10^–6^ mol Ar), or the evolution of 190 gas bubbles of 1 mm in diameter occupying 0.12% of the salt volume (same value also claimed by^17^ for the amount of gas bubbles entrained in their sample) and 1% of the bobber volume. This points to the importance of equilibration during cool-downs to allow for the degassing and to allow for the removal of the bubbles from the salt (dilatometry) or from the bobber surface (hydrostatic method); Ar cover gas and the dilatometry method are probably less sensitive to this effect than He cover gas and the hydrostatic method.

All the sources of error postulated here are well below the 11% variability observed in the experimental data for density. Notable, two of them are high enough to explain the 2% variability in density between the^13^ and^17^ data sets: Li isotopic composition and bubble formation due to temperature-dependent solubility of the cover gas in the molten salt. In this study, the Li isotopic composition is measured (slight enrichment in Li-7 is present), and data shows repeatability both after heating and cooling transients. Transient spikes in mass measurements are observed and attributed to bubble formation; bubble evolution concerns are addressed in this study by bobber shape design to facilitate bubble evolution, time-equilibration, and repeated increasing and decreasing temperature transients to verify repeatability.

## 6 Conclusions

The thermo-physical properties of the molten halide salt 2LiF-BeF_2_ (FLiBe) enable inherent safety features and passive safety systems for fusion and fission nuclear reactors that employ FLiBe, therefore characterization of the thermo-physical properties and the corresponding uncertainty quantification is important for safety analysis of nuclear reactors that utilize FLiBe. The literature referencing the density of molten FLiBe is numerous,^3−6^ but the original sources of data are limited and demonstrate a significant amount of variability. All original data for FLiBe density is compiled in Tables 1, 2, and Figure 1.

We discuss and perform bounding calculations for the possible sources of variability in prior measurements of FLiBe liquid density: salt composition (< 0.6% per 1 mol % BeF_2_), salt contaminants (2%), Li isotopic composition (2%), sample isothermal conditions (0.2%), dissolved gases (< 0.3%), and evolution of bubbles with temperature transients (depending on Ar or He cover gas 0.1 to 0.6% for dilatometry, and 1 to 5% for hydrostatic measurements).

To aid in quantifying thermal expansivity sensitivity to composition, we review and generalize the molar volume additivity prediction for an ideal mixture:
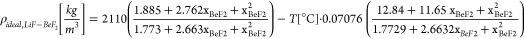
10

To improve this model, measurements are needed for the thermal expansivity of BeF_2_.

The liquid density of FLiBe is measured to be:
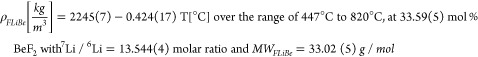
11This result corresponds to an excess molar volume of −1.5% and a thermal expansivity that matches the thermal expansivity predicted by molar volume additivity.

The measurement accuracy of 0.3% for density and 4% for thermal expansivity demonstrated here captures the uncertainty in thermal expansion of the bobber and volume of the bobber, uncertainty in surface tension on the wire and measurement uncertainty for temperature and mass. The York method is utilized for data fitting, to capture error propagation in both the dependent variable (temperature) and the independent variable (density), and the average linear fit residual is 0.1%. The scale is calibrated in the glovebox with NIST-traceable standards and the bobber volume at room temperature is calibrated with NIST-traceable liquid density standards; one of the thermocouples is calibrated with a dry-block thermocouple calibrator.

The dominant sources of uncertainty are the bobber volume uncertainty (0.15%), the mass measurement uncertainty (0.2%), and possibly the wetting angle of the salt on the wire (<0.3%). We postulate that the occasional noise and < 0.2% deviation from linearity is due to the formation of gas bubbles on the bobber surface, related to temperature-dependence of gas solubility. Repeatable results for heating and cooling runs provide confidence that bubble effects are well managed in this experimental set-up. Up to 0.14% increase in thermal expansivity can be attributed to the aluminum contaminant that is likely from the thermal insulation of the oven. With careful attention to experimental design, it is likely possible to further improve the accuracy of density and thermal expansivity measurements. These are the first measurements of the liquid density of FLiBe that report error analysis and that measure the liquid composition before and after density measurements.

## Declaration of Competing Interest

At the time at which the article is published, some of the authors of this manuscript have interests in or relationships with entities that are commercializing molten salt technology. The content of this manuscript or the direction of the research presented herein was not influenced by these entities, nor by the authors’ relationships with these entities.

## Contributions

R.S., R.V., and L.C. proposed the study and designed the experimental test plan. R.V. and L.C. performed the density measurements. L.C. performed the DSC measurements. R.V., S.M. and E.S. analyzed the density data and performed the error analysis. E.S. and R.S. proposed and performed the regression method and the statistical analysis. S.M. and R.S. interpreted the elemental analysis data. R.V. and S.M. provided the literature background. S.M. and R.S. developed the review of the ideal-mixture treatment. R.S. developed the discussion of the sources of error variability and the introduction and validated the data reduction and error analysis. R.S., R.V., S.M., and E.S. wrote the article and contributed to data curation and visualization. R.S.: supervision and funding acquisition.

## Supporting Information Available

The supporting information are available and include the dataset collected for this manuscript, the time-dependent raw data collected for temperature and mass measurements, the literature review of the density measurements of FLiBe, the thermal expansivity from our dataset and from literature data, the density and the thermal expansivity (from literature) as function of the molar fraction of BeF_2_, and the elemental analysis of the FLiBe samples by MC-ICP-MS and SF-ICP-MS.

## Notes

^#^ R.V. and S.M. contributed equally to this paper.
